# Implementing Evidence-Based Dementia Care Interventions: The CMS GUIDE Model and Beyond

**DOI:** 10.1093/geroni/igaf122.827

**Published:** 2025-12-31

**Authors:** David Reuben, Christine Ritchie

## Abstract

There is high need for access to evidence-based dementia care interventions. Use of such services can contribute to higher quality health care, improved clinical outcomes, and cost savings. Multiple strategies have been implemented, many in response to the 2024 launch of the CMS payment demonstration project, the Guiding an Improved Dementia Experience (GUIDE) Model. For health systems participating in GUIDE, efforts are focused on supporting their successful operations or initiating new dementia care programs. The National Dementia Care Collaborative (NDCC) aims to promote dissemination of evidence-based comprehensive dementia care to both GUIDE participants and health care organizations ineligible for GUIDE. The Best Programs for Caregiving (BPC) online database is a resource dedicated to linking health systems, community-based organizations and family caregivers to evidence-based interventions proven to support family caregivers of people living with dementia. Work at Indiana University related to comprehensive dementia care has adapted related approaches to address delirium and critical illness care, and to support navigation during diagnostic processes related to mild cognitive impairment. The objectives of this session are to explain the goals and outcomes of the NDCC, BPC, and work at Indiana University to address multiple chronic conditions, and to show how these efforts can contribute to dementia care implementation. Participants in the session will learn strategies for how health systems and community-based organizations can increase access to evidence-based dementia care while integrating it with other health care and services to improve overall care quality.

